# Detection of *Cystoisospora suis* in faeces of suckling piglets – when and how? A comparison of methods

**DOI:** 10.1186/s40813-018-0097-2

**Published:** 2018-09-19

**Authors:** Anja Joachim, Bärbel Ruttkowski, Daniel Sperling

**Affiliations:** 1 Institute of Parasitology, Department of Pathobiology, University of Veterinary Medicine Vienna, Veterinaerplatz 1, A-1210 Vienna, Austria; 2 CEVA Santé Animale, 10 avenue de la Ballastière, 33500 Libourne, France

**Keywords:** Piglets, Coccidia, *Isospora suis*, Methods, McMaster, Faecal scoring, Carbol-fuchsin, Autofluorescence

## Abstract

**Background:**

*Cystoisospora suis* is the causative agent of porcine neonatal coccidiosis, a diarrheal disease which affects suckling piglets in the first weeks of life. Detection of oocysts in the faeces of infected animals is frequently hampered by the short individual excretion period and the high fat content of faecal samples. We analysed oocyst excretion patterns of infected piglets, evaluated different detection methods for their detection limit and reproducibility, and propose a sampling scheme to improve the diagnosis of *C. suis* in faecal samples from the field using a protocol for reliable parasite detection.

**Results:**

Based on a hypothesized model of the course of infection on a farm, three samplings (days of life 7–14-21 or 10–15-20) should be conducted including individual samples of piglets from each sampled litter. Samples can be examined by a modified McMaster method (lower detection limit: 333 oocysts per gram of faeces, OpG), by examining faecal smears under autofluorescence (lower detection limit: 10 OpG) or after carbol-fuchsin staining (lower detection limit: 100 OpG). Reproducibility and inter-test correlations were high with (R^2^ > 0.8). A correlation of oocyst excretion with diarrhoea could not be established so samples with different faecal consistencies should be taken. Pooled samples (by litter) should be comprised of several individual samples from different animals.

**Conclusions:**

Since oocyst excretion by *C. suis*-infected piglets is usually short the right timing and a sufficiently sensitive detection method are important for correct diagnosis. Oocyst detection in faecal smears of samples taken repeatedly is the method of choice to determine extent and intensity of infection on a farm, and autofluorescence microscopy provides by far the lowest detection limit. Other methods for oocyst detection in faeces are less sensitive and/or more labour- and cost intensive and their usefulness is restricted to specific applications.

## Background

Detection of coccidial infections in domestic animals including pigs can be necessary in a variety of cases. In post mortem examinations of dead piglets, stages of *Cystoisospora suis*, the most important species of coccidia in pigs [1] can be found in histological sections and impression smears (e.g. [2]). This can be helpful in cases of prepatent infections and to determine the extent of pathological changes in relation to parasite infection. As for other enteropathogens, the detection of stages in faeces is a frequent routine to determine an infection in a litter or a herd (usually in relation to clinical signs - in case of coccidiosis, diarrhoea and poor weight gain – or to determine the status of animals as oocyst shedders to estimate the extent of environmental contamination by clinically healthy carriers. In some cases, the efficacy of control strategies is evaluated by determining oocyst excretion after intervention, usually in experimental studies (cf. [3]). Drug resistance has been described for anticoccidial drugs in chicken and recently also in pigs [4, 5] and evaluation of treatment efficacy by faecal examination in the field may also become important in mammalian host species including piglets.

In suckling animals, several issues need to be taken into consideration to accurately determine infection in a litter or a herd. We evaluated sampling schemes and compared the different methods available for the detection and quantification of *C. suis* in piglet faeces and propose a methodology for reliable detection of the parasite in a herd and to evaluate treatment efficacy in cases where treatment failure is suspected.

## Results

### Course of excretion and diarrhoea and sampling time point

**Fig. 1:**
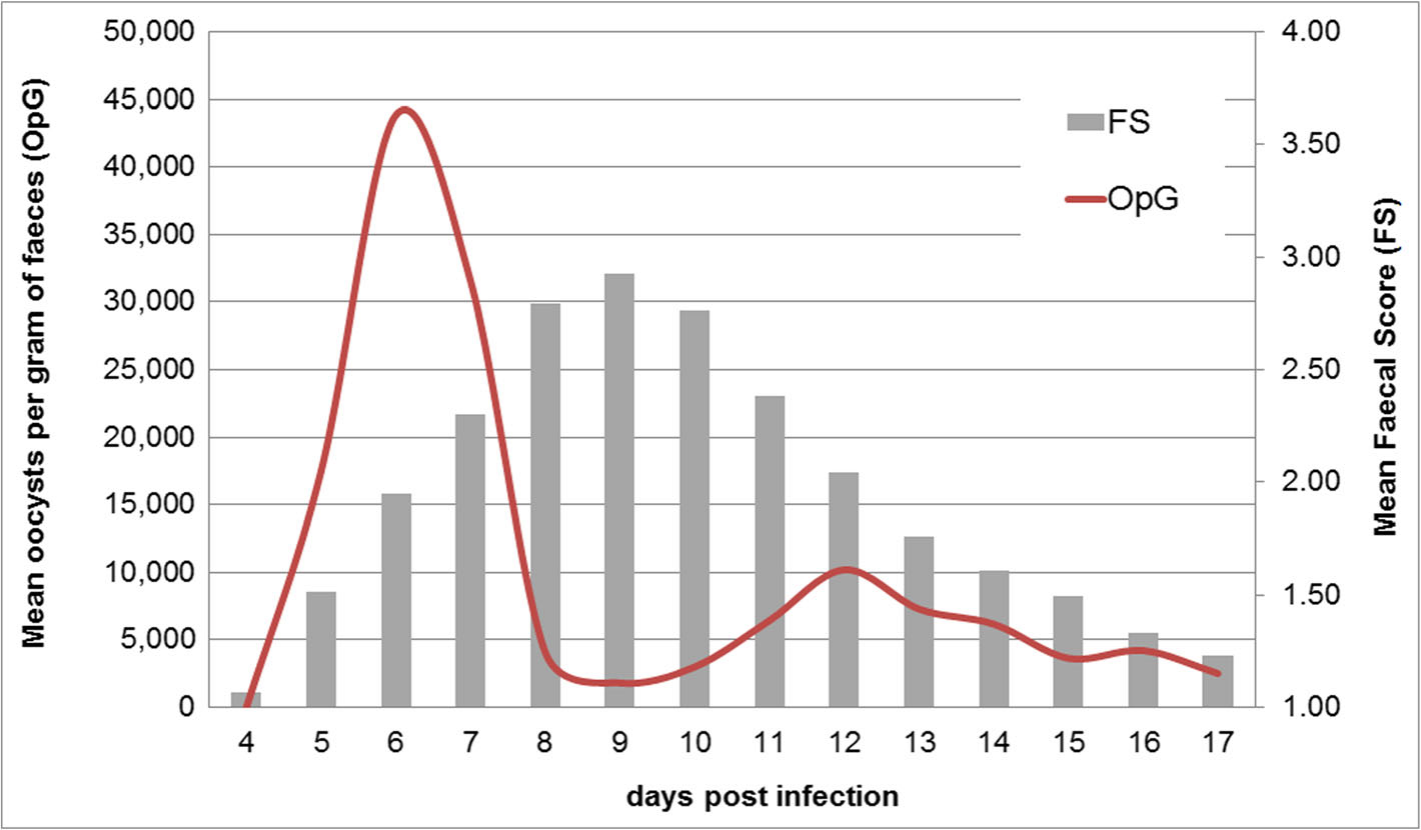
Course of *C. suis* infections; *n* = 117 piglets from different infection trials, adapted from [8]

**Fig. 2:**
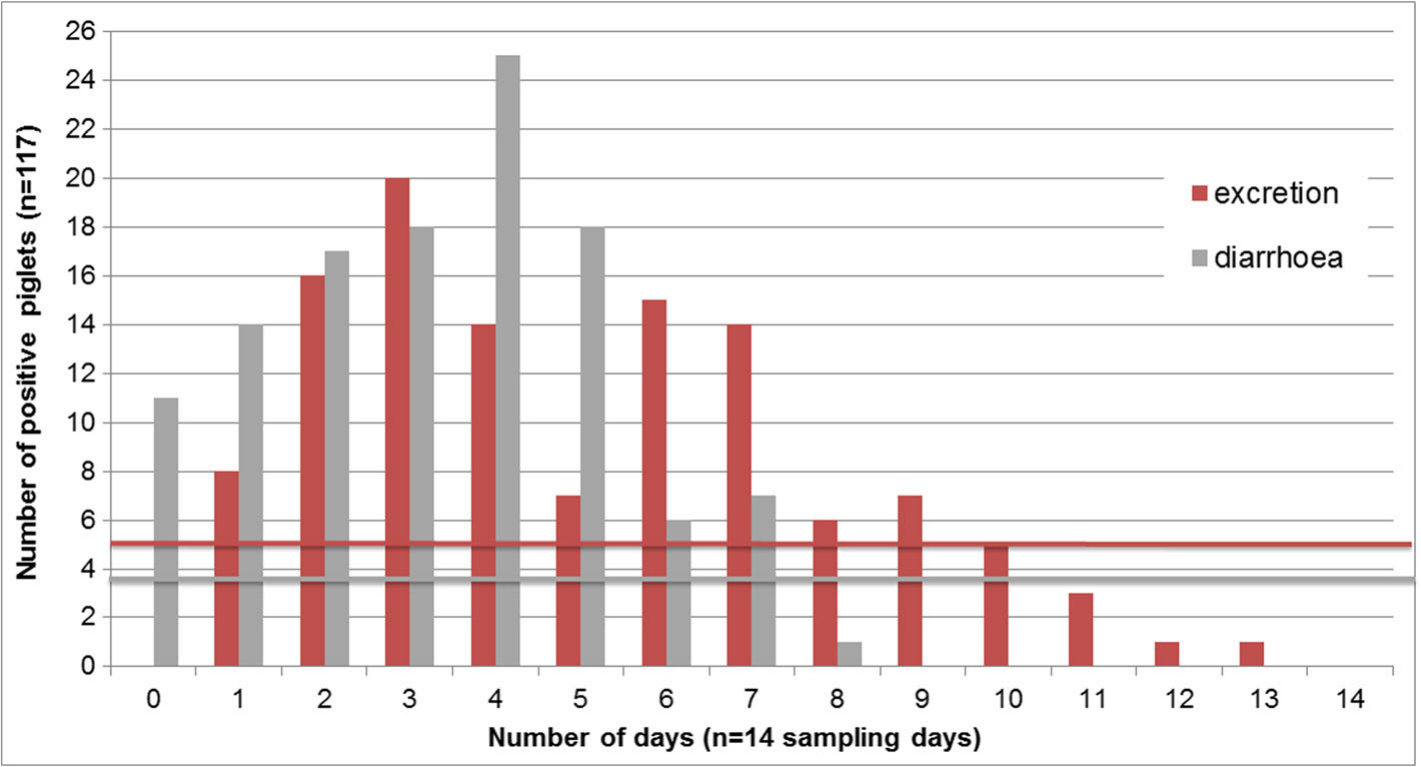
Number of oocyst excretion days and diarrhoea days over a sampling period of 14 days (4–17 days post infection; n = 117 piglets); adapted from [8]. Horizontal bars: mean values (excretion: 5.1 days, diarrhoea: 3.6 days)

**Fig. 3:**
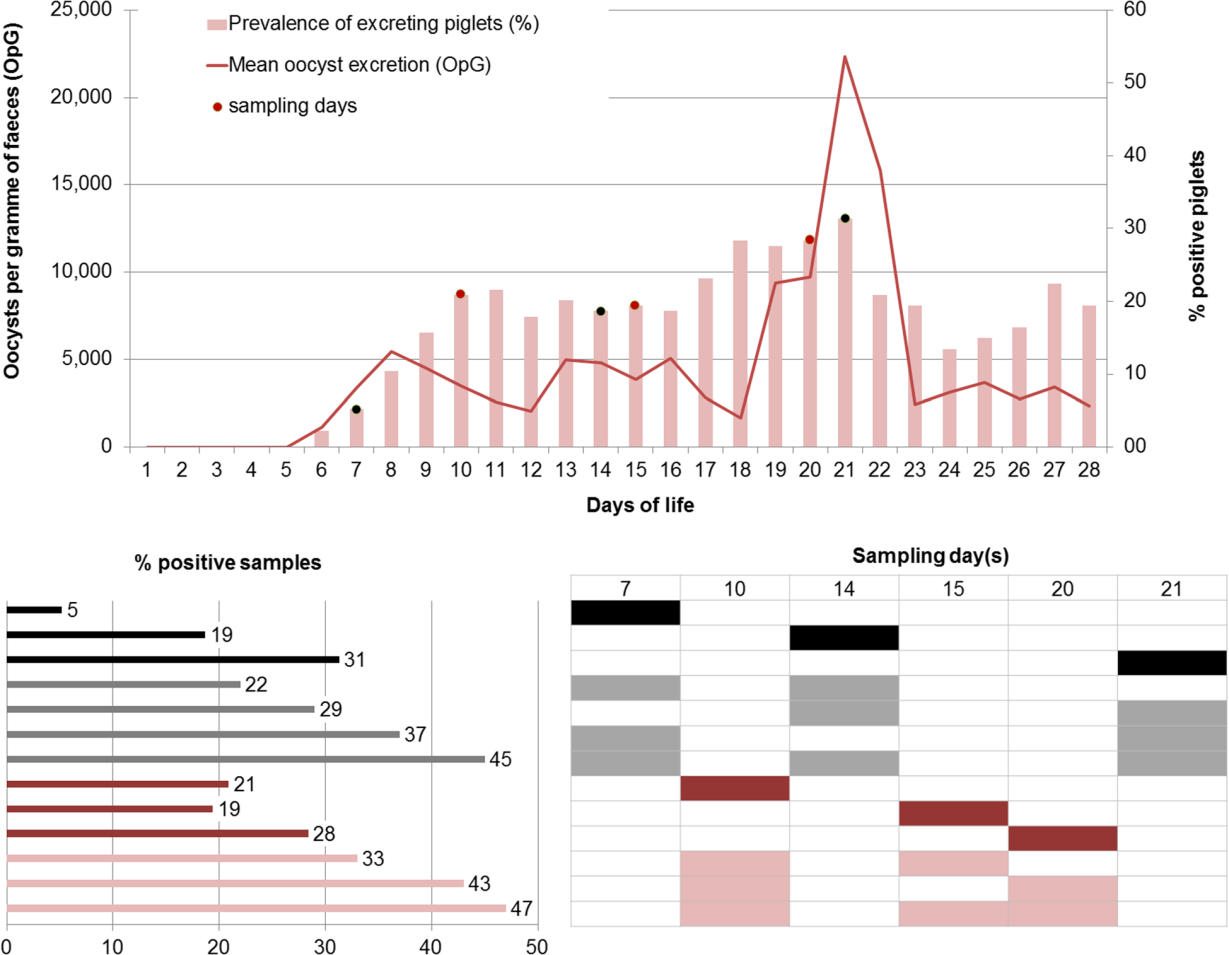
In a model of excretion including *n* = 134 piglets over a period of 28 days with infections between the 1st and the 23rd day of life (with a calculated first excretion day on the last day of sampling) where all piglets excreted at least for 1 day (cumulative excretion rate = 100%; mean excretion days: 4.4, minimum = 1 day, maximum = 13 days; adapted from Joachim et al., 2014), single sampling of individual piglets on different days returns 5–31% positive samples; repeated sampling yields 22–49% positive samples

**Fig. 4:**
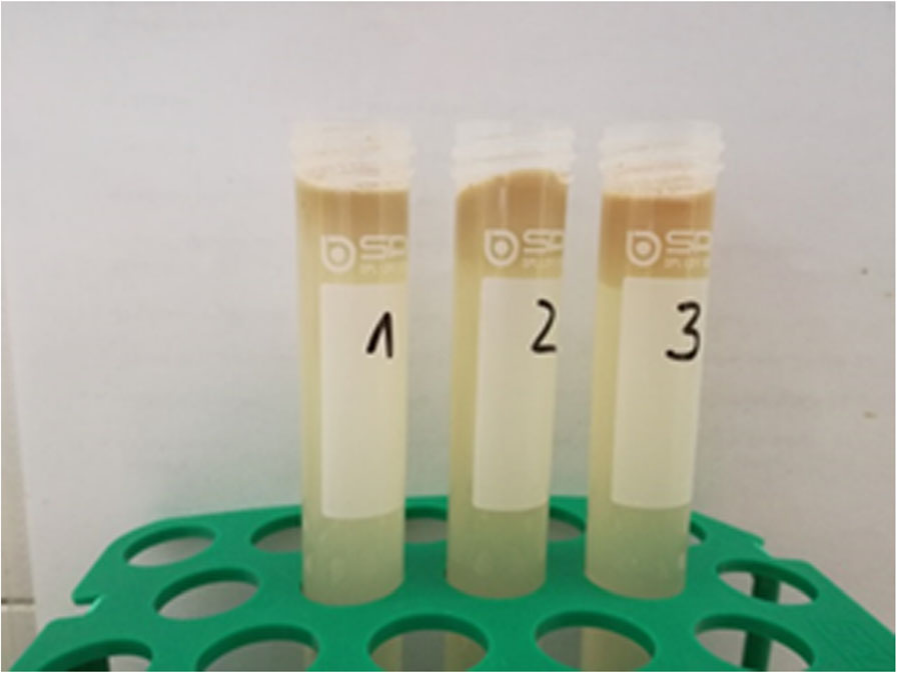
Lipid plugs formed on top of the flotation solutions (1: Sheather’s modified sugar solution, 2: sugar-salt solution, 3: sugar-salt solution + detergent; for details see Materials and Methods) preventing the removal of any parasite objects from the surface

Typically, individual animals show a biphasic excretion pattern upon infection with a steep onset at the beginning of patency, usually five to 6 days after infection (Fig. 1). Excretion can be observed for one to 10 days (median: 5 days) but can be longer in single animals. Similarly, diarrhoea lasts for two to 5 days (median: 4 days) in most animals after experimental infection but can be prolonged in single piglets and is poorly correlated with excretion (Fig. 2). With such a short duration of acute illness and parasite shedding, it can be difficult to determine infections in individual piglets. In a model assuming that all piglets become infected and excrete oocysts for at least 1 day during the suckling period, the prevalence on any 1 day of sampling still never exceeded one third of the animals (Fig. 3). Since it is unknown when infection in the field takes place in individuals or litters, repeated sampling increases the detection rates (Fig. 4). As diarrhoea and excretion are only weakly correlated and do not occur simultaneously (Fig. 1; [6]) a preference for collection of semi-liquid or liquid (diarrhoeic) samples is not indicated.

### Detection of oocysts

After flotation by centrifugation parasitic objects could not be removed from the surface as all centrifugation tubes showed large fatty plugs on top of the flotation medium (Fig. 4) which prevented access to the oocysts. The plugs were removed and treated like faecal smears but could not be stained as crystals of sugar and/or salt interfered with the staining (not shown).

**Fig. 5:**
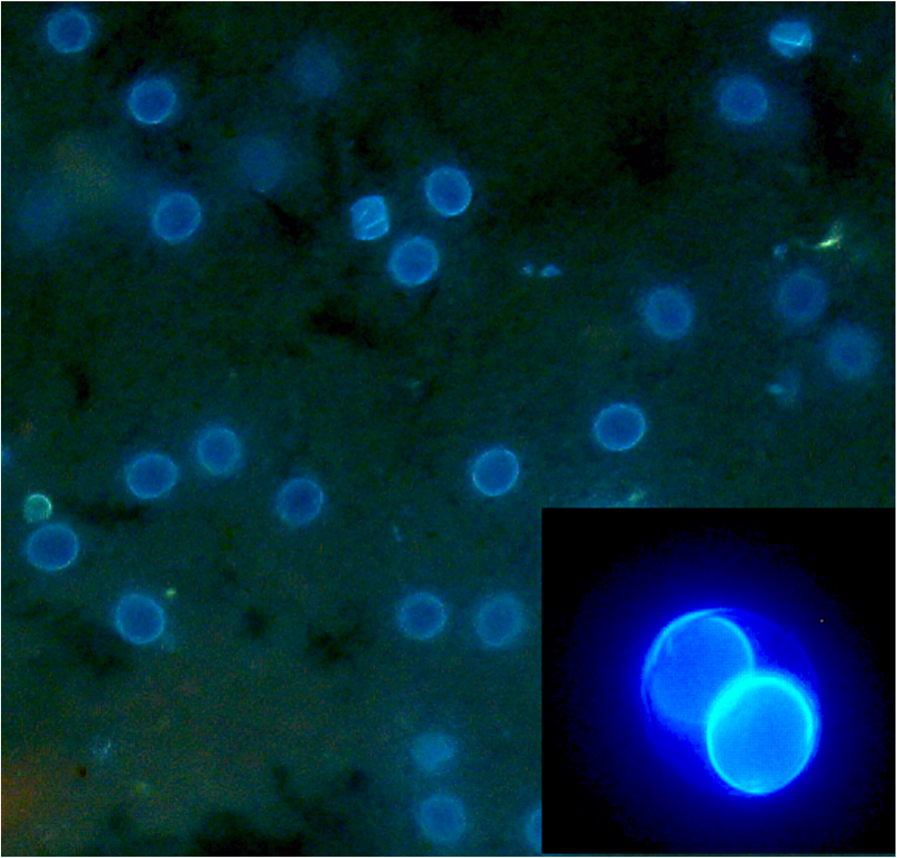
Autofluorescence of unsporulated (large mage, 200× magnification) and sporulated (small image, 600× magnification) oocysts of *C. suis*

Autofluorescence of oocysts could be observed as the emission of blue light form the oocyst (and in sporulated oocysts the sporocyst) wall (Fig. 5). Oocysts of *C. suis* are 18 × 20 μm in size and can easily be differentiated from Eimeria oocysts by their roundish appearance, their thin, smooth wall and, after sporulation, by the number of sporocysts [1].

**Fig. 6:**
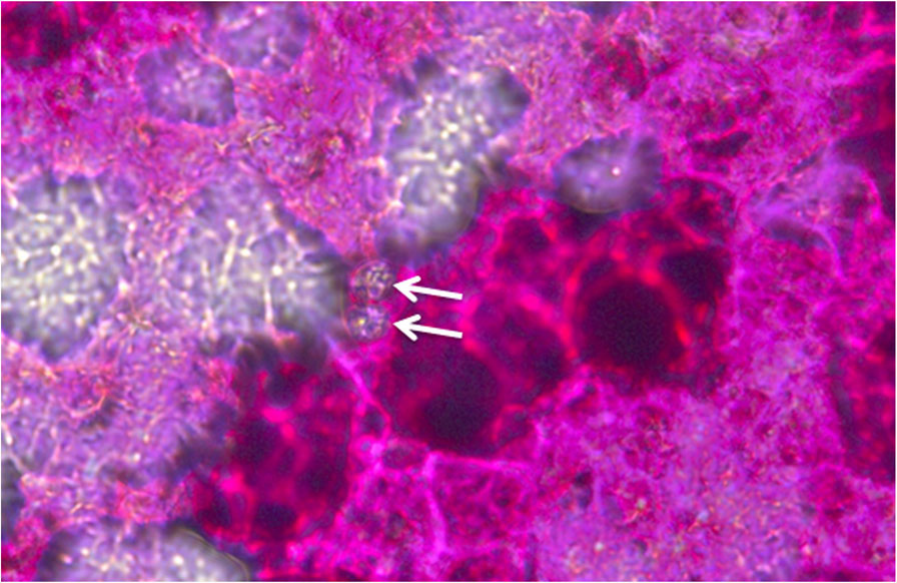
Staining of thin faecal smears for detection of *C. suis* oocysts with carbol-fuchsin. Magnification: 200×. Arrows: unstained sporulated oocysts

In stained smears, oocysts (which usually remain unstained against a coloured background) were detectable only in thin smears (dilution 1:10 compared to those for autofluorescence) but not in thick ones. In unstained samples no oocysts could be visualised under the light microscope. The staining protocol with carbol-fuchsin was the most convenient and the fastest and clearly showed colourless oocysts against the red background (Fig. 6). Counterstaining, e.g. with methylene blue, which is often used to visualised oocysts of cryptosporidia (e.g. [7]), was not necessary for *C. suis* oocysts due to their larger size compared to cryptosporidia (details not shown).

When carbol-fuchsin staining was compared to autofluorescence, all samples were positive for autofluorescence but only 30% were positive after carbol-fuchsin staining, and 43% of the samples that were McMaster positive were also positive upon carbol-fuchsin staining. In firm faeces the detection rate in carbol-fuchsin strained samples was higher (39%) than in loose faeces (28%). The latter also contained fewer oocysts on average (1748 vs. 8288 mean OpG).

### Quantification of oocysts

As outlined above, oocysts can be evaluated semi-quantitatively in faecal smears. Counting in a counting chamber is, however, more accurate since an exact amount of faecal matter can be used irrespective of the faecal consistency. Using the sugar-salt flotation solution in combination with filtration, oocysts could be suspended for floatation in the McMaster chambers. We have adapted this method to small amounts of faeces for the determination of OpGs in individual samples (standard amount of faeces 0.5 g; [6, 8]) or subsets of pooled samples from several individuals in a litter.

**Fig. 7:**
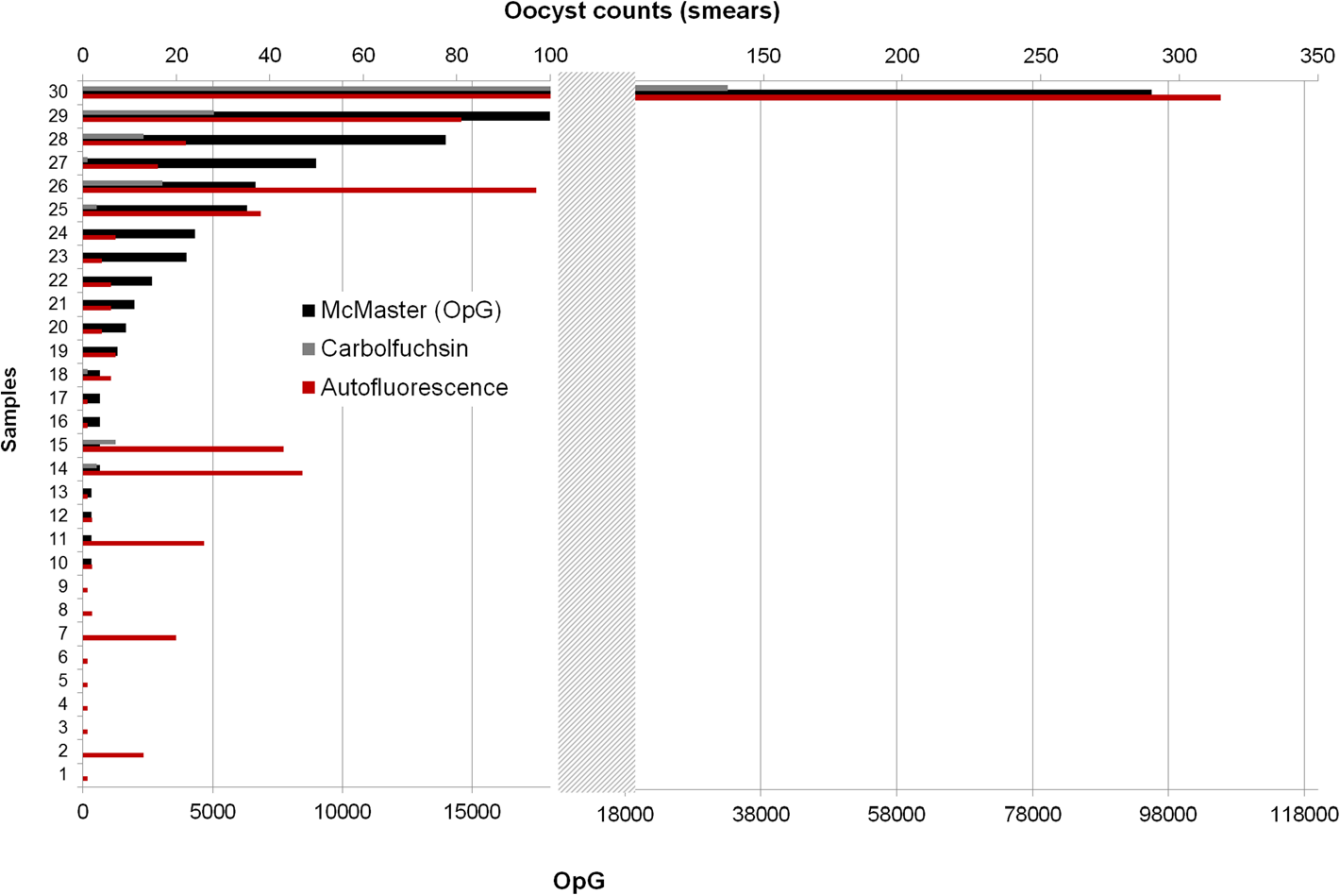
Comparison of McMaster counting (given as OpG) and oocyst counts in smears examined by autofluorescence or light microscopy after carbol-fuchsin staining

The correlations between OpG values and oocyst counts in smears were high with R^2^ = 0.90 for autofluorescence and R^2^ = 0.98 for carbol-fuchsin staining (and R^2^ = 0.97 for autofluorescence v.s. carbol-fuchsin staining), but the mean oocyst counts were considerably higher in the samples examined by autofluorescence (mean: 28.1) than in the carbol-fuchsin-stained samples (mean: 8.5). The calculated lower cut-off for detection of oocysts is 10 OpG for autofluorescence, 100 OpG for the carbol-fuchsin staining (since it requires a 1:10 dilution in comparison to autofluorescence-based examination of faecal smears), and 333 OpG for McMaster. Consequently autofluorescence had the highest percentage of positive samples and the highest absolute oocyst counts (Fig. 7).

**Fig. 8:**
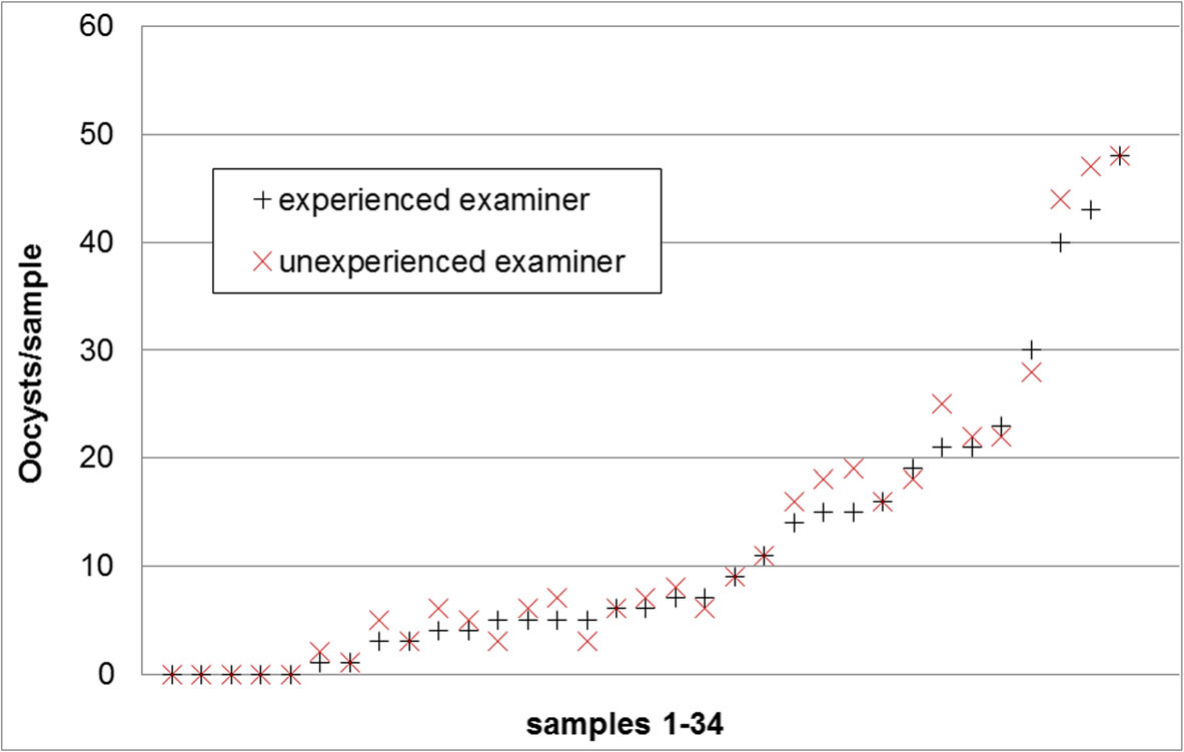
Comparison of absolute oocyst counts by 2 different examiners, an experienced and an unexperienced one of the same faecal smear examined by the autofluorescence method

Comparison of oocyst counts in faecal smears (only positive samples) showed that 28.6% of these had a count > 50 by both examiners, and the inexperienced examiner evaluated two samples (4.1%) as above the cut-off of 50 oocysts (experienced examiner: counts: 46 and 49 oocysts). For the counted oocysts in samples < 50 oocysts (*n* = 35) the correlation (Pearson) was 0.993 (Fig. 8).

**Fig. 9:**
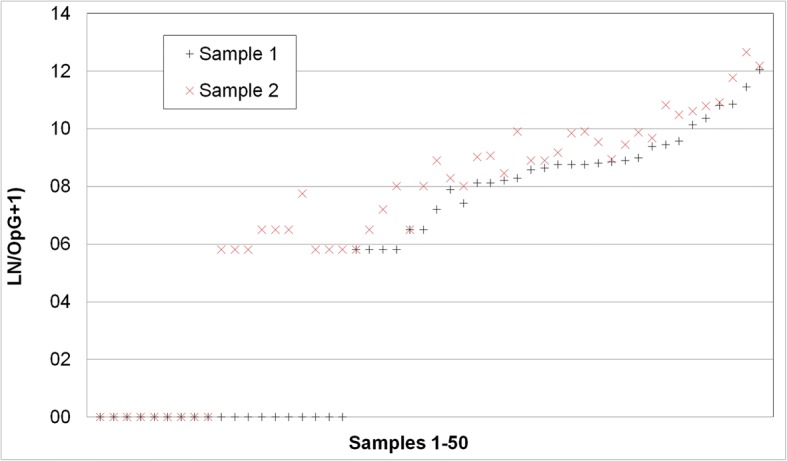
Comparison of OpG counts from 2 independent preparations of the same faecal sample. For better visualisation the higher value was always defined as sample 1 and the lower value as sample 2

Examination of two separately prepared McMaster counts from 50 samples (2 preparations/sample) showed a correlation of 0.852 (Pearson’s correlation coefficient) between sample 1 and sample 2 (Fig. 9). Out of 50 samples, 18% were negative in both examinations, 20% were positive in 1 out of 2 examinations (with counts of 1 or 2 oocysts in the positive samples except one sample were seven oocysts were counted). Of the 31 samples that were positive in both examinations, 11 had OpG values between 333 and 3333 (low OpG), 13 and OpG of > 3333–10,000 in the first count (medium OpG) and 9 had an OpG > 10,000 (max 313,000; high OpG). Only two of the samples that were positive in both counts had identical OpGs (333 and 667, respectively). The deviations were highest in the low-OpG group with a mean of 3.2× (max: 9.0×) and similar in the medium- (mean 2.2-fold, max 5.0-fold) and high-OpG group (mean 2.1-fold, max 4.0-fold).

**Fig. 10:**
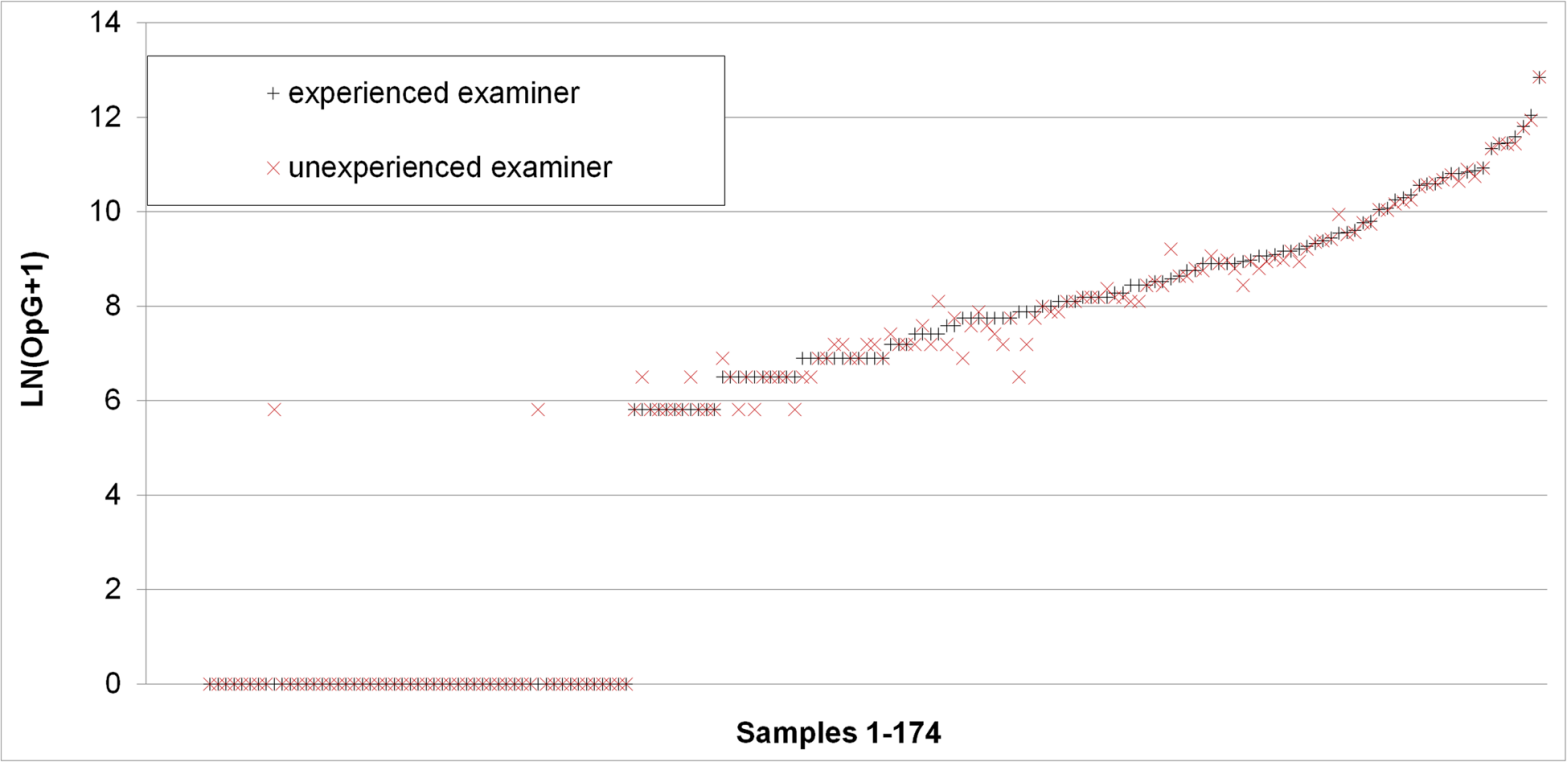
Comparison of OpG counts by 2 examiners, an experienced and an unexperienced one of the same McMaster preparation

Comparison of count results of the same sample by two examiners (*n* = 175 McMaster samples) showed that 65.5% of the samples were evaluated as negative by both examiners and 33.3% were evaluated as positive by both. 1.5% were considered positive (single oocyst counts in each sample) by the unexperienced and negative by the experienced examiner. The correlation of OpGs was very high with 0.998 (Fig. 10).

## Discussion

Lately, anecdotal reports of reduced efficacy of toltrazuril treatment and the first confirmed resistance case are pointing at *C. suis* as a “re-emerging” cause of diarrhoea in suckling piglets, and we aimed to encourage swine practitioners to include this parasite into the regular diagnostic panel irrespective of the treatment history, and we propose a sampling scheme to optimise detection in conjunction with evaluated methods of appropriate detection levels.

### Patterns of excretion and diarrhoea – When to take samples

In most *primo* infections of piglets with *C. suis*, excretion shows a sudden onset, often accompanied by changes of faecal consistency. To determine the correlation between the onset of excretion with diarrhoea, field and experimental studies were evaluated [8–10] but no clear pattern could be determined. In experimental infections the correlation between quantitative oocyst excretion and faecal consistency was weak [6], probably due to the diluting effect of diarrhoea with larger amounts of faeces. It is therefore not advisable to take primarily samples from piglets wit diarrhoea, as such samples may contain few oocysts. Clinically, neonates are most severely affected while infections of older suckling piglets usually display few clinical signs and only low oocyst shedding [11–13]. Older pigs are largely refractory to infection and excretion and diarrhoea are very unusual unless temporary immunosuppression occurs, e.g. in a case of acute viral infections, like in a herd of Swiss fatteners reported lately [14]. Sows usually show low shedding rates with few oocysts [15] which is in part supposedly due to acquired immunity, but also attributed to the pronounced age resistance in *C. suis* infections [12]. Therefore, sampling sows to determine infection on a farm is frequently unsuccessful and samples must be taken from suckling piglets. Prepatency of *C. suis* is four to 5 days so sampling piglets of younger age will not yield a positive result.

Since excretion is usually short (4–5 days on average in the evaluated experimental settings) it may be necessary to sample litters repeatedly to reliably detect oocysts on a farm to confirm the presence of the parasite, or to sample a sufficient number of animals to evaluate treatment success. Even under the assumption that all piglets become infected and shed oocysts before weaning (which might not be the case when infection pressure is low) with a single sampling, the detection rate will not exceed one third of samples, while sampling three times (at 7, 14 or 21 days or 10, 15 and 20 days of age) detects almost half of the positive piglets. Assuming that in a litter all (or almost all) piglets become infected within 1 week, it is possible to reliably detect the parasite in a litter when it is sampled at least twice, and on farms when samples are taken at three different time points. The number of samples to be taken varies with the size of the herd, but examining samples pooled by litter from a maximum of 30 litters (in herds with > 30 sows) will return sufficiently reliable results [15–17].

### Detection and quantification of oocysts in faeces

Several methods of detection have been published that can be applied; however, many of them are not suitable for routine diagnostics. Molecular tools have been used to detect and differentiate stages in faeces with high sensitivity and specificity [18–21] but the processing of samples for DNA extraction from tough oocysts is time-consuming and the high costs of the assay are still prohibitive for routine examination.

Although oocysts are often present in high numbers in individual samples, detection by concentration before microscopic examination can be hampered by the high content of fat in suckling piglets’ faeces (and especially in cases of steatorrhea as described for cystoisosporosis; [22, 23]), which can both prevent detection of oocysts by flotation and impede correct diagnosis in smears as lipid droplets may be taken for unsporulated oocysts. Concentration of oocysts from faecal material of suckling piglets can be problematic since the high fat content may lead to aggregation of a lipid layer with enclosed oocysts on top of the flotation solution after centrifugation. Several modifications of standard protocols are described in the literature. The most common flotation medium for *C. suis* oocysts is Sheather’s sugar solution or modifications of it [24–30]. In our hands, however, none of the applied flotation solutions, even with the use of detergent, could prevent the formation of fat plugs. An alternative to remove most of the fat in piglet faeces is the use of Percoll® in an additional sedimentation step. Percoll® (GE Healthcare) is a density gradient separation medium of low viscosity, low osmolarity and low toxicity. It has been used as flotation solution for *C. suis* in piglet faeces with good success [31] but it is expensive and can be replaced by the cheaper sugar-salt flotation medium. It is, however, most suitable for concentration of oocysts from faeces by sedimentation for further processing of oocysts, e.g. for flotation (Joachim and Ruttkowski, unpublished data).

Some authors prefer the faecal smear with staining over the flotation concentration for reasons stated above [32, 33]. Detection in smears under light microscopy as suggested in earlier works [32, 34] is of poor sensitivity and specificity [35]. However, when autofluorescence is used both can be improved considerably [35]. Upon UV excitation, the walls of the oocysts (and in sporulated oocysts those of the sporocysts) emit a bright blue light that greatly facilitates detection. This phenomenon called autofluorescence has long been known to occur in oocysts of different coccidia [36–39] and is presumably due to tyrosine which is cross-linked in the oocyst wall [40].

Autofluorescence microscopy requires the use of a fluorescence microscope with suitable filters that are standard only in larger laboratories, but the running costs for material and manpower are lower than that of any concentration technique and it is superior to them in sensitivity [41].

If fluorescence equipment is not available faecal smears can be stained by various methods. Carbol-fuchsin staining is quick and easy and can aid the detection of oocysts in faecal smears [33]. Other staining protocols involving auramine O, Löffler’s methylene blue, Lugol’s solution, May-Grünwald or Gentiana violet have been proposed [33, 42] as useful and Ziehl-Neelsen and safranin staining were recently described for the detection of human *Cystoisospora belli* oocysts in faeces [43], carbol-fuchsin is easiest to apply and the contrast was sufficient to detect oocysts in smears, although autofluorescence is still far more sensitive.

For quantification of oocysts in faecal material, counting of oocysts in a McMaster chamber is standard [20, 23, 44, 45]. Since the confounding effect of lipid droplets can also occur in this method (albeit without centrifugation) Henriksen and Christensen suggested the use of saturated sugar solution instead of saturated sodium chloride [46]. A further modification was suggested by the same authors using gauze filtration of faeces in this sugar-salt solution before counting [47]. We have adapted the original method [6, 16] for the use on individual piglet samples (0.5 g/sample) but it can be used for larger amounts as well.

When counting of oocysts in smears by autofluorescence was compared to McMaster counting, there was a high correlation between the two methods; however, autofluorescence had a tendency for higher relative values so it must be assumed that with the McMaster method oocysts are lost during preparation or do not flotate. Also, McMaster counting has a cut-off value of 333.3 OpG while autofluorescence is much more sensitive with 10 OpG. Autofluorescence and McMaster counting both showed a high correlation between examinations by two different persons, with a slight tendency for higher values in case of the unexperienced examiner, especially in the autofluorescence microscopy, so false positive counts must be considered when staff is trained in this method. When McMaster counting was run on two independent preparations from the same sample, samples with a low OpG frequently gave back differing qualitative results and showed a high variation in the quantitation. When values for the area under curve of the OpG are statistically evaluated this has to be taken into account, especially when the results for this parameter are not supported by other parameters for oocyst excretion comparison between different groups in a treatment trial (ref. [46], for statistical evaluation of oocyst excretion in intervention studies).

For the sensitive qualitative and semiquantitative evaluation of oocysts in piglet faeces, autofluorescence appears the method of choice as previously shown [40]. It is easy and quick to do and can also be used to determine the extent of excretion when applied to samples from individual piglets, e.g. in prevalence studies [6]. If the equipment is not available, carbol-fuchsin staining according to Heine [48] can be applied. It is well correlated to autofluorescence, albeit less sensitive, but it could reliably detect oocysts in sample with an OpG of 6000 and more. Since during peak excretion average OpGs reach 45,000 this method can still be sued to determine the presence of oocysts on a farm when repeated sampling is applied (see above).

In summary, under field conditions oocyst of *C. suis* can be detected on a farm by repeated sampling of individual piglets and examination of pooled samples per litter. Oocyst detection can be accomplished by detection in faecal smears with autofluorescence or, with relatively lower sensitivity, after carbol-fuchsin staining, and can be judged semi-quantitatively. Determination of OpGs by adapted McMaster counting is usually applied under experimental conditions and for specific applications, such as an in vivo faecal oocyst reduction test to evaluate treatment efficacy [49]; however, this requires precise knowledge about the extent and intensity of infection and the course of infection on the farm in question.

In the light of recently reported reduced treatment efficacy for toltrazuril, *C. suis* should be re-considered as a diarrhoeal pathogen. We hereby suggest diagnostic procedures (repeated sampling and examination of faecal smears by autofluorescence or carbolfuchsin staining) that can be used to determine both the presence and the extent of infection on a farm to provide diagnostic tools for the evaluation of treatment efficacy and semi-quantitative determination of the infection rates in a herd.

## Methods

### Determination of prevalences of oocyst shedding at different time points

We used a subset of data obtained from experimental infections of neonatal piglets (*n* = 134 piglets) from previous studies with known infection days, onset and duration of excretion as determined by daily sampling and qualitative and quantitative oocyst excretion [8]. Over a period of 14 days (4th to 17th day after infection, i.e. during the patent period) the course of oocyst excretion and faecal consistency was determined for 117 piglets. Assuming that piglets can become infected for the first time from the first day of life until weaning we modelled 23 subgroups (*n* = 5–8 randomly allocated animals/group with a total of 134 modelled individuals) with *primo* infections on the 1st to 23rd day of life. This time span was chosen because the minimum pre-patent period for *C. suis* is 3 days (e.g. [24, 44, 50] but most piglets will not start excretion before the 5th day after infection [6, 8, 30, 51, 52]. The last day before weaning was assumed to be the 28th day of life so piglets older than 23 days could become infected but do not excrete oocysts before weaning. Different infection doses were not taken into consideration as these do not seem to have an appreciable influence on the onset and duration of excretion [8].

### Detection methods for *C. suis* in faecal samples

We examined different methods of faecal concentration and faecal smears according to different protocols and compared their detection limit as well as the time needed to prepare and examine the samples. Faecal samples (firm or pasty consistency) of piglets with known status of excretion (from experimental studies) were pooled, mixed well and examined in parallel using different methods.

To concentrate oocysts from faecal samples by flotation different media were used: Sheather’s sugar solution [25], sugar-saturated sodium chloride solution [44], or sugar-saturated sodium chloride solution with detergent (1/100 volume of household dishwasher detergent). For each sample 1.5 g of faeces were mixed with approx. 14 ml of flotation solution, filtered through sieve and funnel and centrifuged at 600 x *g* for 10 min.

Faecal smears were prepared from the same material (approximately 0.1 g/sample) and used natively for autofluorescence or stained with different methods prior to microscopic examination. For autofluorescence, the samples were covered with a glass cover slip and examined at 100× magnification under UV light (excitation wavelength: 340–380 nm; [35]).

Staining of faecal smears was carried out by mixing 0.1 g of faeces with 2–3 drops of the respective solution (1% carbol-fuchsin, 5% malachite green, 1/ nigrosine or 1% light green; ref. [48, 53]), and spreading the mix on a glass slide (a thick smear of 90% of the total amount and a thin smear with 10% of the volume further diluted 1:10 in tap water were prepared). Dried stained smears were examined by light microscopy as described above. In some cases the presence of oocysts had to be verified under 200× magnification.

To compare the detection limit of autofluorescence and carbol-fuchsin staining, 30 samples of piglets from experimental studies were examined as described above; 18 of them had a firm consistency, 12 were pasty to liquid. Nine had a low amount of oocysts (and were negative by McMaster counting, see below) and 21 had a countable oocyst per gram of faeces (OpG) value (median: 1665 OpG, maximum: 95571 OpG). Oocysts were counted in the smears to evaluate correlations with OpG values.

### Quantification of oocysts

**Fig. 11:**
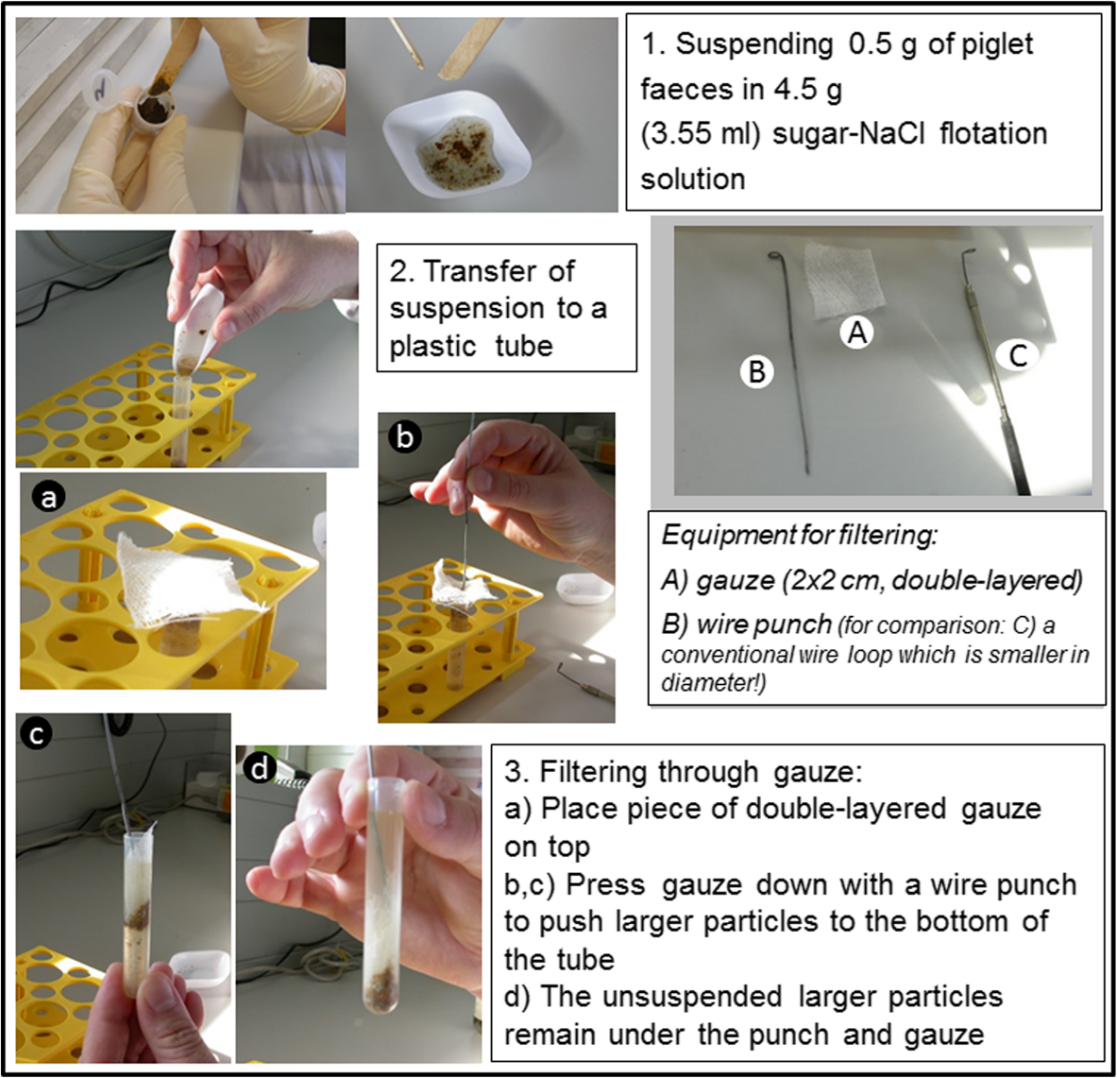
Preparation of individual piglet samples for isolation of *C. suis* oocysts in flotation medium

Oocyst numbers can be determined in faecal smears, but to obtain more accurate data on the number of oocysts per gram of faeces (OpG) modified McMaster methods are routinely used. Faecal matter (0.5 g) was mixed thoroughly with 4.5 g (3.55 ml) of a salt-sugar solution (50 g of sucrose added to 100 ml of saturated sodium chloride solution prepared from 400 g of sodium chloride ad 1 l of tap water); this suspension is then filtered by pressing a double-layered piece of gauze through the suspension to the bottom of a round-bottom tube (5 ml single-use plastic tube, 12 × 75 mm, round-bottom) using a metal loop (Fig. 11). This prevents the unsuspended lipid matter from floating in the suspension and trapping the oocysts. Immediately after filtering the suspension is further diluted 1:10 (200 μl + 1800 μl of solution) in the salt/sucrose solution, mixed well and filled into the McMaster chamber(s).

For the McMaster counting a cut-off of 666.6 for one field results from the dilution (1:100) and the size of the chamber (150 μl), and the oocysts per gram of faeces are calculated as follows:

$$ \mathrm{OpG}=\frac{\mathrm{X}\ast 100}{0.15\ \mathrm{ml}} $$ or OpG = X ∗ 666.7

with X being the number of oocysts counted in one field. As a standard we calculate two fields/sample. This method was used to determine the OpG values for the first part of the evaluation (see above).

The reproducibility of oocyst counting in autofluorescence and McMaster was evaluated by examination of 49 autofluorescence-positive faecal smears analysed by autofluorescence by two different examiners, 174 McMaster preparations by two different examiners (an experienced and an unexperienced one) and by re-examining samples by one examiner in a second preparation (*n* = 50).

## Conclusion

*C. suis*-infected piglets usually excrete oocysts for short time periods, so the right timing and a sufficiently sensitive detection method are important to determine the presence and extent of the parasite in a herd. This should also be considered in cases of poor efficacy of toltrazuril treatment. Faecal samples should be taken repeatedly to improve sensitivity. Autofluorescence microscopy of faecal smears provides by far the highest sensitivity for oocyst detection. Other methods are less sensitive and/or more labour- and cost intensive and their usefulness is restricted to specific applications.

## Abbreviations

*C. suis*: Cystoisospora suis
FS: Faecal score
OpG: Oocysts per gram of faeces
